# Supplementary material for: Piloting Siyakhana: A community health worker training to reduce substance use and depression stigma in South African HIV and TB care
Source: PLOS Glob Public Health. 2024 May 7;4(5):e0002657. doi: 10.1371/journal.pgph.0002657 (PMC11075908; doi:10.1371/journal.pgph.0002657)
Supplement: S1 File — (DOCX) [file pgph.0002657.s001.docx]

**S1 File. ENACT Roleplay Staff Training**

**Roleplay Instructions for Participant:**

You are a CHW providing support for people, including those who have HIV and TB. The person you are about to meet was referred to you through a community outreach program and you have gone to their house to meet them for the first time. This is your first time meeting them. You have no further information about this person. You have approximately 10 minutes to learn about the person and what they are dealing with and engage in a supportive manner. Please use the full ten minutes with this patient, even if you think you might ask for help from a supervisor in the community.

**Roleplay Instructions for Staff Member Actor:**

***Pre-Training Scenario:***

Several years ago, you started having a hard time. This started after your father died in an accident. You and he had had a difficult relationship and a big fight soon before he died meant that you were not speaking much at the time of his death. You struggled with feelings of guilt after this and started to go out drinking with friends sometimes so you could get away from home and from thinking about your father. At first, this helped you stop thinking about your problems for a little while. But it also made you feel worse (physically and mentally) the next day, which made it hard to get to work and to clinic appointments and made your mother and your sister worry. Over time you gradually started drinking every night and beginning earlier and earlier in the evening. You stopped doing things that you used to enjoy, like gardening and hanging out with friends. Recently you have sometimes been drinking during the day, which led to you getting fired from your job for showing up drunk. You are anxious about not having taken your medications for the past several months but you are also anxious about returning to the clinic because the nurses may be upset with you for not having come to recent appointments. Sometimes you think it might be easier for everyone if you were not around.

***Post-Training Scenario:***

Several years ago, you started having a hard time. This started after you were injured when you were working at a restaurant. You had a hard time with pain after that and had to quit your job to recover. You realized that drinking alcohol could help lessen the pain for a bit and would help you sleep. Over time your alcohol consumption started increasing, until the point that you are having around 5 drinks every day. This has made you feel worse in the morning, but you were already struggling with motivation to get up in the morning since you don’t have to be at work. Sometimes you wish you wouldn’t wake up at all. You have started cooking and doing household chores less and found yourself getting irritable with your children and friends more. Thinking about not having been to the clinic for a few months for your medication makes you feel guilty, but you can’t find the energy to travel there.

***General Notes for Staff Actor:***

Overall tone/presentation.

- Do not provide your name or any other personal information unless asked to do so by the participant
- Look off to the side or look down when speaking about your problems; take long breaths/sighs and pauses between talking; speak softly
- Do not tell the participant right away that you are depressed and have problems with alcohol. Start with something more general like, *“I have been having a hard time…”*
- Do not share about feelings or emotions unless the participant asks you how you are feeling
- If the participant uses close-ended questions (e.g., *“Do you,” “Did you,” “Can you,”* etc.,) respond with short yes/no responses
- If the participant uses open-ended questions (e.g., *“Please tell me about that,” “please share with me,” “How did that…”*, etc.,) respond with more detailed answers.
- If the participant uses technical terms (such as acronyms, medical terms, or other terms that a patient may not be familiar with) ask *“What does that mean?”* to see if the participant can describe it in lay language.
- If the participant asks for feedback on what they are telling you, reply *“Some of the suggests are helpful, but some seem too hard for my situation.”* Then ask if there are other options or activities.
- If the participant asks about problems or difficulties in your life, say *“I don’t have the energy to do anything – this is all my fault.”*

Confidentiality.

- At some point in the ten-minute roleplay, mention concerns about confidentiality. For example, you may say something like *“my sister is home, I am worried she might hear us,”* or *“are you going to tell anyone these things that I tell you?”*
- If the participant does not mention any limits to confidentiality when they discuss confidentiality, reply, *“does that mean there are absolutely no times when you will tell anyone anything?”*

Suicidality.

- At some point in the ten-minute roleplay, tell the participant, *“Sometimes I think it’d be easier if I didn’t wake up in the morning.”*
- If the participant asks if you want to die or kill yourself, say, *“No, I wouldn’t do that. I want to live for my children.”*
- If the participant asks if you have ever tried to harm your kill yourself before, say, *“No, I have never tried to do that.”*

Unrealistic Expectations

- At some point in the ten-minute roleplay, ask the participant questions such as, *“Will meeting with you help me get a job?”*
- If the participant asks about your goals, say *“I must make a lot of money soon* (or something else likely unrealistic) *if I will make things up to my mother and sister.”*
