# Supplementary material for: Piloting Siyakhana: A community health worker training to reduce substance use and depression stigma in South African HIV and TB care
Source: PLOS Glob Public Health. 2024 May 7;4(5):e0002657. doi: 10.1371/journal.pgph.0002657 (PMC11075908; doi:10.1371/journal.pgph.0002657)
Supplement: S1 Table — (DOCX) [file pgph.0002657.s003.docx]

**S1 Table. Training Components**

| Module | Component | Training Day |
| --- | --- | --- |
| Psychoeducation (Information) | Stigma | 1 |
|  | HIV & TB | 1 |
|  | Depression | 1 |
|  | Substance Use | 1 |
|  | Culture | 1 |
|  | CHW Role | 1 |
|  | Confidentiality | 2 |
|  |  |  |
| Self-Care Skills | Mindfulness | 1 |
|  | Values | 2 |
|  | Reflecting on own background | 3 |
|  | Balancing life activities | 3 |
|  |  |  |
| Evidence-Based Patient Skills | Problem-Solving Therapy | 1 |
|  | Motivational Interviewing | 2 |
|  | Nonjudgmental Communication | 2 |
|  |  |  |
| Lived Experience Video | Depression | 1 |
|  | Substance Use | 1 |
