# Supplementary material for: Piloting Siyakhana: A community health worker training to reduce substance use and depression stigma in South African HIV and TB care
Source: PLOS Glob Public Health. 2024 May 7;4(5):e0002657. doi: 10.1371/journal.pgph.0002657 (PMC11075908; doi:10.1371/journal.pgph.0002657)
Supplement: S2 Table — (DOCX) [file pgph.0002657.s004.docx]

**S2 Table. ENACT Clinical Competencies**

| Item | Competency |
| --- | --- |
|  |  |
| ENACT 1 | Non-Verbal Communication |
| ENACT 2 | Verbal Communication |
| ENACT 3 | Explains and Promotes Confidentiality |
| ENACT 4 | Rapport Building and Self-Disclosure |
| ENACT 5 | Exploration and Normalization of Feelings |
| ENACT 6 | Demonstration of Empathy, Warmth, and Genuineness |
| ENACT 7 | Assessment of Harm and Developing Response Plan |
| ENACT 8 | Connect to Social Functioning and Impact on Life |
| ENACT 9 | Explore Client’s Explanation for Problem |
| ENACT 10 | Involvement of Family and Significant Others |
| ENACT 11 | Collaborative Goal Setting |
| ENACT 12 | Promotion of Realistic Hope for Change |
| ENACT 13 | Incorporation of Coping Mechanisms and Prior Solutions |
| ENACT 14 | Psychoeducation with Local Terminology |
| ENACT 15 | Elicitation of Feedback |
