# Supplementary material for: Piloting Siyakhana: A community health worker training to reduce substance use and depression stigma in South African HIV and TB care
Source: PLOS Glob Public Health. 2024 May 7;4(5):e0002657. doi: 10.1371/journal.pgph.0002657 (PMC11075908; doi:10.1371/journal.pgph.0002657)
Supplement: S3 Table — (DOCX) [file pgph.0002657.s005.docx]

**S3 Table. ANOVA Comparing SDS Scores Across Vignettes and Assessments.**

| Predictor | df | F | p | Partial η^2^ |
| --- | --- | --- | --- | --- |
| Participant | 16 | 2.65 | 0.005** | 0.47 |
| Time | 1 | 3.30 | 0.08 | 0.06 |
| Vignette | 1 | 46.10 | <0.001*** | 0.49 |
| Time*Vignette | 1 | 7.69 | 0.008** | 0.14 |
| Residuals | 48 |  |  |  |
