# Supplementary material for: Piloting Siyakhana: A community health worker training to reduce substance use and depression stigma in South African HIV and TB care
Source: PLOS Glob Public Health. 2024 May 7;4(5):e0002657. doi: 10.1371/journal.pgph.0002657 (PMC11075908; doi:10.1371/journal.pgph.0002657)
Supplement: S4 Table — (DOCX) [file pgph.0002657.s006.docx]

**S4 Table. T-Test Comparing total ENACT Scores at Different Assessments.**

| Comparison | n | M | (SD) | t | (df) | p | d |
| --- | --- | --- | --- | --- | --- | --- | --- |
| Pre-Training | 12 | 27.33 | (4.14) | -3.34 | (11) | 0.007** | 0.97 |
| Post-Training | 12 | 31.42 | (3.94) |  |  |  |  |
