# Supplementary material for: Piloting Siyakhana: A community health worker training to reduce substance use and depression stigma in South African HIV and TB care
Source: PLOS Glob Public Health. 2024 May 7;4(5):e0002657. doi: 10.1371/journal.pgph.0002657 (PMC11075908; doi:10.1371/journal.pgph.0002657)
Supplement: Attachment — Submitted filename: Siyakhana.docx [file pgph.0002657.s007.docx]

- Can you be more concrete about how SU and depression related stigma is related to loss of follow-up? For instance, how much more likely are patients to drop out and express SU and depression stigma as a reason for their loss of follow-up vs. people who are lost to follow-up for other reasons?
- Why were these two NGOs selected specifically?
- How did you select the roleplay new patient? How was their training done?
- Since you’re using number between parenthesis in the methodology, i.e. “According to the Link and Phelan stigma framework, mental health and SU stigma may exist 150 among CHWs, when (1) CHWs label their patients who have SU and/or depression symptoms as 7 151 different and attach negative stereotypes to them, and (2) separate these patients (‘them’) from 152 other patients and themselves (‘us’)”,

this type of listing could be easily confused with the referencing, despite using brackets for the references and parenthesis for your listing of the steps taken. I suggest listing your steps using another format, such as

“According to the Link and Phelan stigma framework, mental health and SU stigma may exist 150 among CHWs, when a.) CHWs label their patients who have SU and/or depression symptoms as 7 151 different and attach negative stereotypes to them, and b.) separate these patients (‘them’) from 152 other patients and themselves (‘us’),

- Maybe you should provide a bit more information for how you integrated Link and Phelan’s stigma framework and the sIMB-CIM
- One of the assumptions to us ANOVA is that your data is normally distributed. I suggest adding a sentence clarifying which normality test you used to test for normality to ensure all statistical steps were taken.
- My apologies, as non-native English speaker, I am struggling to find sense to the word “vignette” in your paper. It is my understanding vignette is more often referred to presenting a clear description, sketch, or picture of something, so I’m thinking that when you use vignette, you mean something like “participant characteristics”. Please reconsider your phrasing of this word for something simpler or closer to what you mean, as it could be confusing for some of your readers.
- In fig.1 all training days have 17, which is your total number. Training days should have the individuals who were trained for each day, summing up to 17 instead.
- Since the training was done in English, it would be healpful to detail how you controlled or minimized language barriers during the training, and/or desirability bias when the participants were answering their surveys, specially the post-training assessment.
