# Supplementary material for: Piloting Siyakhana: A community health worker training to reduce substance use and depression stigma in South African HIV and TB care
Source: PLOS Glob Public Health. 2024 May 7;4(5):e0002657. doi: 10.1371/journal.pgph.0002657 (PMC11075908; doi:10.1371/journal.pgph.0002657)
Supplement: Attachment — Submitted filename: Reponse to Reviewers.docx [file pgph.0002657.s008.docx]

**EDITOR:**

**1. Kindly place the person before the disease and therefore replace terminologies such as 'TB patient' and 'HIV patient' with 'people with TB' and 'people living with HIV/AIDS' or similar wherever applicable.**

Thank you for catching this. We have updated the language appropriately throughout the text.

**2. While this study is a pilot as stated it the design Type 1 hybrid implementation study seems a bit premature for this study. You may consider (though not necessarily) an 'intervention study' or 'quasi-experimental feasibility pilot' as alternatives.**

Thank you for this suggestion. We agree that it may be premature to call this study a Type 1 hybrid implementation study, as it was conducted to inform a subsequent randomized Type 1 hybrid implementation study. Based on your suggestion, we now refer to it as a “pilot feasibility study” (line 110). We also clarify that this is a pilot study to inform a future Type 1 hybrid effectiveness-implementation study on line 429.

**3. Kindly indicate if your study is powered adequately to assess effectiveness.**

The main purpose of this study was to refine the implementation of a CHW training for a larger clinical trial. Consequently, the present study was not adequately powered to assess effectiveness. We have clarified throughout the text that all effectiveness findings should be viewed as preliminary and have added text about power to lines 290-292.

**4. The study states short term effectiveness was assessed- however, the assessments for effectiveness were done only pre and immediately post intervention and not beyond, also the term preliminary effectiveness has been used elsewhere. Do consider the use of uniform terminology throughout the manuscript.**

Thank you for this comment. We have now updated the manuscript to uniformly state “preliminary effectiveness” instead of “short-term effectiveness.” We have also clarified throughout that we were comparing pre-training and post-training scores.

**5. Please consider depositing your data in an open access repository- or alternatively indicating how this data may be made available to those interested.**

Thank you for this suggestion. We are actively engaged in a conversation with our ethics board (South African Medical Research Council Human Research Ethics Board) about whether this is allowable without reconsenting our participants. We are also researching options for which open access repository to use; if deemed allowable by our ethics board, we will plan to deposit the present data alongside our subsequent larger trial data. In the meantime, we have indicated that this data is available to those interested upon written request to the study principal investigators (lines 307 – 308).

**6. I request you to kindly address the comments from the reviewers that have been stated below.**

Thank you for your thoughtful feedback. Our responses to specific reviewer comments are below, which we believe have substantially improved the manuscript.

**Reviewer #1:**

**Stigma is an important area for evidence-based interventions among people living with HIV. Your manuscript reads well and you have explained about the trainings and the tools used in the study well. Please find my comments below.**

Thank you for this feedback. Please find responses to your comments below.

**39-42: Here the patients with SU and depression are people living with HIV-TB co-infection? If yes, then authors may give some figures to show how much problem is SU/ depression in this group.**

Thank you for this suggestion. Based on this comment, we have now added more information supporting that SU and depression are common among people living with HIV, TB, and HIV/TB co-infection in South Africa. Specifically, we now include:

[lines 73 – 83]: “Concurrently, it is estimated that South Africa has high rates of problem substance use (SU) [4­­-7] and depression [8], including among people living with HIV and TB [9-14]. For instance, a recent South African study examining patients with TB in primary care found that 83% screened positive for a psychiatric diagnosis, with depression among the most common diagnoses, and 43% screened positive for a SU disorder. Over half of these patients were concurrently living with HIV, with similar rates of psychiatric and SU diagnoses identified in these patients [13]. In other studies, rates of depression have been estimated to be as high as 41% among people with HIV [9] and 64% among people with TB in South Africa [14]. While there is limited data on the rates of SU among people with HIV and TB, previous work from our team found nearly one-third of people initiating ART had unhealthy alcohol use [11], and 88.4% of people initiating TB treatment had unhealthy alcohol use or smoked illicit drugs [12].”

**44: Instead of working, re-engaging would be a better word here since we are dealing with those patients who have dropped out of care.**

Thank you for this suggestion. We agree that *re-engaging* is a better word here than *working* and have updated the line accordingly (line 44).

**60: Please add ‘long-term’ before the word engagement.**

We have added long-term before engagement (now line 61).

**61-63: Please revise the sentence.**

We have revised the sentence and hope it is now more clear:

[Lines 62 – 67]: “Tuberculosis (TB) is a leading cause of death among people living with HIV, including in South Africa, the country with the greatest number of people living with HIV and one of the highest rates of HIV/TB co-infection [3]. As adherence to both HIV medication (i.e., antiretroviral therapy (ART)) and TB medication is required to prevent death in those co-infected [3], strategies to retain and re-engage people in care for both is critical.”

**75: Authors may use mental health ‘conditions’ as used in the WHO mhGAP.**

Thank you for this suggestion. We have updated the word “concerns” to “conditions” (line 95).

**96-97: Authors may remove repetition of “preliminary effectiveness.”**

We have removed this repetition (line 116).

**104: It will be helpful for the readers if authors can provide a brief description of roles and responsibilities of CHWs and if they are based in the community or health facility.**

Thank you for this comment. The CHWs typically work in the community, visiting the homes of patients. However, while most of their work occurs in the community, they are linked to a clinic, and get their patient assignments through this clinic. Common tasks for CHWs include delivering patient medications, providing basic health education, and following up with patients who have disengaged from chronic care. We have added this information to the manuscript:

[Lines 123-127]: “CHWs are generally linked to a specific health clinic and visit the homes of patients with various health concerns and needs. Common CHW tasks include delivering medications to patients and providing daily observed therapy for TB, providing patients with basic health education, and following-up with patients with HIV and/or TB who have disengaged from HIV or TB care.”

**Study procedures: Please mention who conducted these trainings? What was their expertise in conducting such trainings. It has been mentioned towards the conclusion in the manuscript. It will be good if some information is provided in the methods section as well.**

Thank you for this suggestion—we agree that it makes sense to include who conducted the trainings in the Methods section. We have now clarified that all trainings were delivered by two psychological counsellors and one peer counsellor, all of whom were employed as study staff, under the Study Procedures section.

[Lines 166 – 168]: “All trainings were delivered by two bilingual psychological counsellors registered with the Health Professions Council of South Africa, and one bilingual peer counsellor, all of whom were employed as study staff.”

**162-63: “Specific content of the training was informed by formative qualitative interviews..” Can authors list the contents in a table?**

Thank you for this suggestion. We have now added a new supplemental table (Table S1), which lists all training content. Please see lines 209 – 210 for reference to this supplemental table.

**194: What were the two time points? Before and after training? Please specify.**

The two timepoints were pre-training (i.e., immediately before the first day of training) and post-training (i.e., immediately after the training). We have added a sentence stating this on lines 232 – 233 and have added “(pre- to post-training)” when introducing and discussing outcome measures.

**262: What is the meaning of ‘believed’ here? Not clear.**

We have changed the wording from “believed” to “endorsed having” to make it clearer (lines 318, 388).

**326-28: In table 1 authors have reported 90% of CHWs had a family member with SU/depression. They are already having contact with people with lived experience of SU. In spite of this the baseline stigma scores for SU was high. How do the authors explain this?**

Thank you for this comment. We now discuss this in the manuscript:

[Lines 387 – 394]: “Although most people endorsed having a family member with depression or SU, baseline SU stigma levels were quite high. One explanation for this was we asked people whether they had anyone with depression or problem SU in their family, rather than differentiating the two. Thus, it is possible most people had someone with depressive symptoms, not symptoms of SU disorder, in their family. Second, contact in the context of a training aimed at increasing understanding of these conditions and teaching skills for working effectively and empathetically with such patients may be more beneficial for reducing stigma than just any type of contact.”

**342-49: This is very important to highlight. I am glad authors have discussed this in this manuscript.**

Thank you – we appreciate this feedback.

**Reviewer #2:**

**This is a well written manuscript.**

Thank you. We appreciate this feedback.

**1. In the study setting line 106, could be good to mention the name of the two NGOs that you hard partner with unless they said they should not be mentioned.**

Thank you for this comment. We have now added the names of the NGOs to the Methods section (lines 127 – 128). Additionally, we mention the NGOs in the Acknowledgements section.

**2. Raw de-identified data especially the quantitative should be deposited on the public repository e.g., demographic, pre-post scores, competency, etc. Here is an example of a public space for depositing the data:** [**https://zenodo.org/**](https://zenodo.org/)

Thank you for this suggestion. We are actively engaged in a conversation with our ethics board (South African Medical Research Council Human Research Ethics Board) about whether this is allowable without reconsenting our participants. If deemed allowable by our ethics board, we will plan to deposit the present data alongside our subsequent larger trial data. In the meantime, we have indicated that this data is available to those interested upon written request to the study principal investigators (lines 307 – 308).

**Reviewer #3:**

**Can you be more concrete about how SU and depression related stigma is related to loss of follow-up? For instance, how much more likely are patients to drop out and express SU and depression stigma as a reason for their loss of follow-up vs. people who are lost to follow-up for other reasons?**

Thank you for this comment. Based on your suggestion, we have added some examples of how SU and depression stigma may be related to treatment disengagement to lines 84 – 95. For instance, in qualitative interviews with patients, patients have said that stigma towards depression/SU from providers, including CHWs, has made them not want to return to care due to perceived judgment, criticism, and discrimination. Likewise, patients have reported that they worry about CHWs being the people to screen for or manage common mental health symptoms because of the negative attitudes CHWs have towards mental health. Patients with SU and depression are also more likely to have worse engagement in treatment, and the lack of support and perceived judgment may further reduce motivation and comfort in seeking services. While we are unaware of prior research quantifying the reasons why patients are lost to follow-up, and there are undoubtedly reasons other than depression/SU stigma, these qualitative findings from our prior work and others suggest that stigma toward mental health may be a potentially modifiable factor that contributes to worse engagement in care.

[Lines 84 – 95]: As SU and depression are associated with worse HIV and TB outcomes [1,2], such as more missed visits and greater likelihood of being lost to follow-up [7,8], it is likely that many of the patients who are visited by CHWs for recent disengagement in HIV/TB care experience symptoms of depression and/or SU. Yet, while the CHW role is meant to facilitate care engagement, research suggests that CHW stigma towards SU and depression can act as a barrier to engagement in HIV and/or TB care [19-24]. For example, in previous qualitative work, patients living with chronic conditions like HIV who were experiencing common mental health conditions (e.g., depression, substance use) shared not wanting to return to care because of how providers, including CHWs, treated them [20,21], and worried that CHWs would not be appropriate people to screen for and intervene with common mental health conditions because of CHWs’ negative attitudes towards such conditions [22].

**Why were these two NGOs selected specifically?**

These two NGOs were selected because they employ the majority of CHWs in the areas in which we were working (areas which also have very high rates of HIV, TB, and substance use). We have added this clarification to lines 127 – 131.

**How did you select the role play new patient? How was their training done?**

We have now clarified that the patient in the roleplay was played by one of two staff members who were minimally involved in training, both from the community, familiar with the patient population, and experienced in mental health intervention training. These staff members were trained in how to conduct the role play based on best practices developed by Dr. Brandon Kohrt, who originally developed this method for standardized assessment of non-specialists in task-shared mental health care. Specifically, a US-based team member (ALR) was trained by Dr. Kohrt and his team on role play methods, and she then trained the role play actors. Additionally, we have included a new supplemental file (Supplemental File 1) that presents an overview of the roleplay instructions.

[Lines 150 – 155]: “Participants also completed a brief, videorecorded roleplay where they were instructed to “meet” with a new patient (played by a staff member) who was experiencing symptoms of problem SU and depression. This staff member was minimally involved in the training, was from the community and familiar both with the patient population and mental health intervention training, and was trained in the roleplay methodology by a US-based team member (ALR) based on research best practices [32]. Please see Supplemental File 1 for an overview of the roleplay instructions.”

**Since you’re using number between parenthesis in the methodology, i.e. Line 149-152 *“According to the Link and Phelan stigma framework, mental health and SU stigma may exist among CHWs, when (1) CHWs label their patients who have SU and/or depression symptoms as different and attach negative stereotypes to them, and (2) separate these patients (‘them’) from other patients and themselves (‘us’) …”* This type of listing could be easily confused with the referencing, despite using brackets for the references and parenthesis for your listing of the steps taken. I suggest listing your steps using another format, such as *“According to the Link and Phelan stigma framework, mental health and SU stigma may exist among CHWs, when a.) CHWs label their patients who have SU and/or depression symptoms as different and attach negative stereotypes to them, and b.) separate these patients (‘them’) from other patients and themselves (‘us’)…*”**

Thank you for this suggestion – we agree that how we originally wrote this could be confusing. We have now changed the list to “a)”, and “b)” (lines 182 – 187).

**Maybe you should provide a bit more information for how you integrated Link and Phelan’s stigma framework and the sIMB-CIM**

We have now added more details to how we integrated these two frameworks to inform the training:

[Lines 191 – 197]: “In this integrated model, CHWs would be provided with information to increase understanding of depression and SU and reduce stigma (i.e., accurate psychoeducation on SU and depression; information on referrals and resources in community; information on stigma and why it can be harmful); taught non-judgmental communication skills to decrease stigmatized interactions with patients; asked to reflect on similarities between themselves and patients to decrease stigma towards patients; and taught motivational and problem-solving skills to help patients to better navigate and engage in care.”

**One of the assumptions to us ANOVA is that your data is normally distributed. I suggest adding a sentence clarifying which normality test you used to test for normality to ensure all statistical steps were taken.**

We now clarify that ANOVA and t-test assumptions were checked prior to conducting analyses. For normality, we examined a QQ plot of the models’ residuals and conducted a Shapiro-Wilk test of normality (lines 301 – 303).

**My apologies, as non-native English speaker, I am struggling to find sense to the word “vignette” in your paper. It is my understanding vignette is more often referred to presenting a clear description, sketch, or picture of something, so I’m thinking that when you use vignette, you mean something like “participant characteristics”. Please reconsider your phrasing of this word for something simpler or closer to what you mean, as it could be confusing for some of your readers.**

Thank you for this feedback – we most definitely want to ensure that all readers are able to fully understand our methods, regardless of their first language. In English language psychology research, “vignette” is often used to describe a short narrative (delivered verbally or in text) about a hypothetical character in a particular situation. To keep our description of the Social Distance Scale (SDS) measure consistent with other SDS descriptions and methodology, we are hesitant to change the word “vignette.” However, to clarify what we are describing, we have now added a definition for vignette in lines 238 – 239. We hope this definition will make our methods clearer to all readers.

[Lines 241 – 242]: “…participants were given two case vignettes (i.e., short narratives about hypothetical characters in specific situations)…”

**In fig.1 all training days have 17, which is your total number. Training days should have the individuals who were trained for each day, summing up to 17 instead.**

Thank you for this feedback. In the figure, we meant to connote that all 17 participants attended all days of the three-day training. We have now added a note to Figure 1 that explains this.

**Since the training was done in English, it would be helpful to detail how you controlled or minimised language barriers during the training, and/or desirability bias when the participants were answering their surveys, specially the post-training assessment.**

Thank you for this comment. We now address this in the Discussion section:

[Lines 442 – 445]: “To minimize this risk, study staff who were bilingual in English and other commonly spoken languages (isiXhosa, Afrikaans) were available to help participants when needed. Participants were also reminded that answering honestly would help us better refine the training, that their answers were confidential, and that they would not be penalized or judged for any of their answers.”
